# Major depressive disorders related to Post-traumatic cases in Geriatic Patients with traumatic brain Injuries

**DOI:** 10.1192/j.eurpsy.2026.11393

**Published:** 2026-07-31

**Authors:** N. Syrmos

**Affiliations:** Aristotle University, Thessloniki, Greece

## Abstract

**Introduction:**

Introduction-Post-traumatic stress disorder and major depressive disorders are close mental pathological situations in which the cause is readily identifiable. The two disorders represent distinct constructs and depend on separate biological mechanisms. Both, however, are also related to stress psychopathology

**Objectives:**

Aim-Aim of this study is to present cases with post-traumatic stress disorders cases and major depressive disorders in geriatric patients with traumatic brain injuries.

**Methods:**

Material-Methods-10 geriatric patients are presented. 9 males (90%) and 1 females (10%) Range of age between 68-78 years old . All of theme (10-100%) were evaluated, with clinical and radiological exam(ct,mri) plus with the Diagnostic and Statistical Manual of Mental Disorders (DSM-5) diagnostic criteria, prevalence, and presentation of patients with PTSD.

**Results:**

Results-Good results in 8 cases (80%) and moderate in 2 (20%). Collaboration between various medical disciplines is essential in order to achive optimal results and clarify the theory,the etiology, and treatment effectiveness.

**Conclusions:**

Conlusions- Recommendations regarding effective psychotherapy, neurological,psychiatric and pharmacotherapy, emerging treatment, and accurate management are necessary in order to achieve optimal overall treatment.

**Disclosure of Interest:**

None Declared

